# Automatic Joining of Electrical Components to Smart Textiles by Ultrasonic Soldering

**DOI:** 10.3390/s21020545

**Published:** 2021-01-14

**Authors:** Sebastian Micus, Michael Haupt, Götz T. Gresser

**Affiliations:** 1 German Institutes for Textile and Fiber Research Denkendorf, (DITF), 73770 Denkendorf, Germany; michael.haupt@ditf.de (M.H.); goetz.gresser@ditf.de (G.T.G.); 2 Institute for Textile and Fiber Technologies (ITFT), University of Stuttgart, 70569 Stuttgart, Germany

**Keywords:** smart textiles, wearables, ultrasonic, soldering, joining, integration of electronics

## Abstract

A suitable connection method to automatically produce E-textiles does not exist. Ultrasonic soldering could be a good solution for that since it works with flux-free solder, which avoids embrittlement of the textile integrated wires. This article describes the detailed process of robot-assisted ultrasonic soldering of e-textiles to printed circuit boards (PCB). The aim is to understand the influencing factors affecting the connection and to determine the corresponding solder parameters. Various test methods are used to evaluate the samples, such as direct optical observation of the microstructure, a peeling tensile test, and a contact resistance measurement. The contact strength increases by reducing the operating temperature and the ultrasonic time. The lower operating temperature and the reduced ultrasonic time cause a more homogeneous metal structure with less defects improving the mechanical strength of the samples.

## 1. Introduction

According to current studies, the market volume for intelligent textiles will grow significantly in the coming years. In addition to the current medical and military applications, the usage will expand to the fields of fashion and sports [1].

The challenge is to ensure the usual textile properties for the customer, such as textile feeling, washability [2], wear comfort, and drapability. At the same time, the textile must be able to withstand changing environmental conditions.

However, we currently do not see a real market breakthrough due to the following reasons. One of the reasons for the low market penetration of electronic textiles is the high proportion of manual activities during production [3]. In addition, the high proportion of manual production steps results in high prices, which slow down market growth. Therefore, the German Institutes for Textile and Fiber Research (DITF) have intensively researched on automated contacting processes. In this work we focused on textile integrated insulated wires. These wires are located inside the textile enabling to connect components from both sides. Automated methods for contacting printed circuit boards (PCBs) with insulated wires have not yet been sufficiently investigated in the literature. The manufacturing process typically consists of three steps [4]. First, the wires are automatically uninsulated with a laser. The contacting process starts afterwards, which is investigated in this article. To protect the electronics from environmental influences, the components are overmoulded by an injection molding machine [5]. For this reason, we investigated the mechanical properties of the connections in a peeling test. This allows us to determine whether the connection is strong enough for further production steps.

The challenges in contacting electronics to textiles are as follows. Macroscopically, the structure is very homogeneously produced. None of the sections resemble each other, thus the mechanical behavior of the textiles always differs during processing. This leads to problems in the positioning step between the textile and the electronic. The durability of the contacts is also one of the problems regarding the expected high mechanical stress of e-textiles.

However, there is a challenge due to the processes used so far. Each of these processes has the following difficulties: Mechanical strength (durability of the contacts), fatigue resistance and the implementation on an industrial scale.

### 1.1. Force-Fit Connections

The integration of electronics to smart textiles by force-fit connections needs two steps: The mechanical connection to the textile and the electrical connection to a conductive structure. The function of smart textiles is only guaranteed if both connections are reliable. Force-fit connections have the advantage that they can form both connections without thermal stress on the substrate. Simon et al. [6] developed and integrated a force-fit interconnection and investigated the influence of the applied force and the used strips or yarns on the contact resistance [6]. In the end, the required effort to produce the screwed connections is clearly too high and no automation could be realized.

### 1.2. Form-Fit Connections

Crimp connections are robust, highly reliable, low-cost and fast and easy in processing. Simon et al. [7] developed multi-terminal crimp packages to integrate them into large-area smart textiles. The woven textiles had integrated conductive yarns inside. Therefore, they designed special forming tools. These tools were able to bend eight pins at the same time, so they were able to applicate the crimp terminals much faster compared to with serial production steps [7] These methods did not become generally accepted. Due to the inhomogeneity of textiles, the difficult positioning especially with large textiles and the low adaptability of the tools between different products.

Klink et al. [8] used printed isoplanar and anisotropic conductive adhesive (ACA) flip chip connections for the integration of thin chips into textiles. The printed isoplanar connections showed high resistances and none of the samples failed during a temperature reliability test. These results confirm the thermomechanical stability of the assembly. The ACA contacts with pure copper are susceptible for degradation. The absence of this effect in silver plating indicates that the origin of these failures is not mechanical fatigue but due to the degradation of the copper and the ACA contact interface.

Linz et al. [9] developed a theoretical model of the contact mechanism underlying embroidered contacts. They used the model to identify potential failure mechanism and exposed temperature cycles as a reliability test. Additionally, they encapsulated the contact zones with different thermoset plastics. As a result, they determine a quickly lose in conductivity during their temperature cycle tests. Only the combination out of epoxy adhesive in the contact zones and subsequent encapsulation with hot melt showed good results [9]. 

In the field of Smart Textiles, snap fasteners are often used for ECG measurements. They allow the placement of larger evaluation units on textiles and their reversible removal during a possible wash cycle. Examples for the application can be found in [10,11,12,13,14]. For the integration of miniaturized electronic components such as sensors or actuators this is not an option. Only one connection can be made and a snap fastener usually has the dimensions of a complete sensor.

### 1.3. Substance-to-Substance Bonds

Furthermore, Linz et al. [15] present a new technology to connect conventional electronics with conductive textiles. They introduced nonconductive anisotropic adhesives (NCA) as a reliable method for contacting rigid electronic modules to fabric substrates. They used thermoplastic polyurethane as non-conductive adhesive. The technology brings the PCB and the conductive adhesive in contact. Afterwards, they use load and temperature to melt the adhesive. If the isolation of the textile integrated wire has a lower melting point than the thermoplastic polyurethane (TPU) adhesive, they can also contact wires with insulation. In the following, they used harsher reliability test conditions and more detailed investigations of non-conductive adhesives to confirm the advancement of this technology [15].

Hirman et al. [16,17] focused in their research on connecting SMDs onto textile ribbons by UV curable NCA. The ribbons had silver coated copper threads inside. Even after several washing, temperature and load cycles they dedicated a reliable connection between the electronics and the ribbon [16,17].

Atalay et al. [18] used different stainless-steel and different silver-plated yarns to create a signal transmission lines resistant. Therefore, they used the ultrasonic welding technology. The conductivity of the stainless-steel yarns changed slightly, while the silver-plated yarns were more affected by the ultrasonic welding technology. Furthermore, the electrical resistance of the silver-plated yarns showed huge variations. Thus, they considered to use stainless-steel yarns for the proposed technology [18].

Shi et al. [19] investigated the bonding mechanisms of ultrasonic welding in fabrics. Therefore, they used different natural and synthetic fabrics with and without polyurethane adhesive. They studied the influence of the three important welding parameters: amplitude, welding time and welding pressure. Furthermore, the temperature inside the connection was measured during processing [19].

Leśnikowski et al. [20] welded textile nickel coated, fabric signal lines (TSLs) between non-conducting textile layers by using ultrasonic welding technology. He analysed the usability of ultrasonic welding for the integration of TSLs. Unluckily, the resistance of a direct welded TSL increased [20].

Buechley et al. [21] built up a construction kit for e-textiles, on which they soldered an IC socket to a fabric PCB or metal crimping beads to surface mount LEDs and motors. The resistance of the solder joints increased a bit but remained below 1 Ω even after washing [21].

Kallmayer et al. [22] developed a solution for the integration of passive silicon transponder labels in textiles. These flexible chips can overcome high mechanical load but the integration required new integration technologies because of their dimensions and the possible tolerances between the microelectronic and the textile. As a solution, they soldered the transponders to conductive yarns with a low melting alloy. The connections survived conventional washing process without any failures after encapsulation [22].

Hot bar soldering has several advantages, which makes it particularly suitable for contacting PCBs on Smart Textiles. The process offers the possibility of contacting several contact points on textiles at the same time [23]. Furthermore, pressure and temperature are applied. This allows the conductor and PCB to be fixed to each other. The thermode is usually made of titanium [24] or molybdenum. Furthermore, such a system offers the advantage to use it for curing conductive adhesives. As described by Woznicki [25], hot bar soldering has problems with very small pad pitches. A lot of textile has to be removed to connect single contacts.

Another connection method is conventional piston soldering. It always uses solder with flux. Flux is necessary to activate the surface of the solder joints and to remove impurity layers. Flux in particular removes oxidized metal from the surfaces of the solder joint, it seals out air, thereby preventing further oxidation, and it improves the wetting characteristics of the liquid solder to facilitate amalgamation. It allows the solder to flow nearly in the same way as lead-containing solders, which are forbidden in most applications. Soldering with lead-containing solders is now only permitted under strict regulatory requirements and for special applications. When soldering with flux, the solder is drawn into the strand due to capillary effects. The solidified solder outside the soldering point itself causes an embrittlement of the otherwise very ductile strands. This results in weak spots close to the connection points, which cause the electronics to fail under cyclical load. The difficulty of conventional piston soldering is a high motivation for the investigation of ultrasonic soldering.

## 2. Materials and Methods

### 2.1. Ultrasonic Soldering

**Figure 1:**
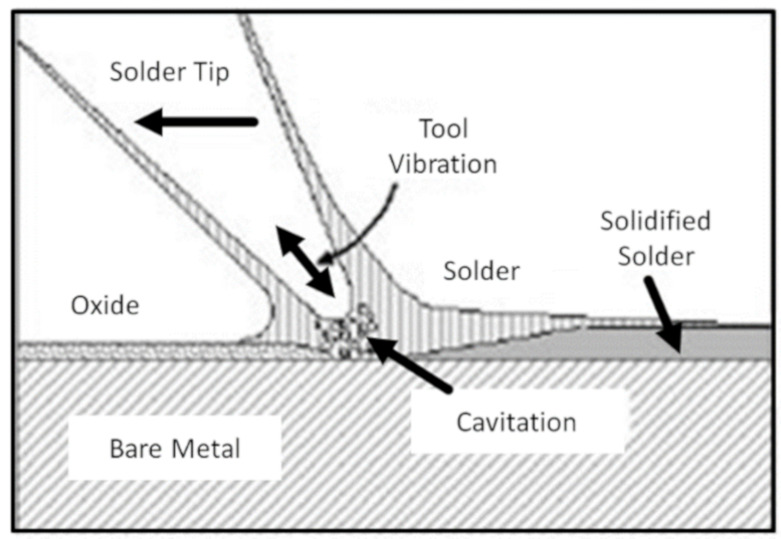
Schematic illustration of the cavitation mechanism in ultrasonic soldering [26].

During ultrasonic soldering, the heat input occurs primarily through the temperature-controlled solder tip (Figure 1). In contrast to ultrasonic welding or wire bonding where the heat energy is generated by ultrasonic friction the temperature-controlled soldering tip melts the solder and the additional ultrasonic movement of the tip removes boundary layers on the surface, which ensures solderability without flux. The ultrasonic excitation leads to cavities in the liquid solder, which remove impurities (impurity layer) from surfaces similar to the principle of ultrasonic cleaning. Two main principles are common in ultrasonic soldering:

■ Similar to dip soldering: The solder bath is heated and excited with ultrasonic. However, this application is not suitable for textiles as the textile comes into contact with solder and melt.
■ Similar to piston soldering—soldering iron with an additional ultrasonic generator: The frequency power and temperature can be adjusted.

Special solder alloy without flux must be used for ultrasonic soldering since soldering materials with flux inhibit the transmission of ultrasound. Due to the absence of flux, less solder penetrates into the strand though the wick effect. As a result, the strand becomes less brittle and the entire textile retains higher flexibility. In addition, the entire process can be easily automated and scaled. Due to years of use in the electronic industry, the soldering machines are well controllable and available in different dimensions. The contact points have a high strength and good electrical conductivity.

### 2.2. Experimental Setup

A warp-knitted textile tape was used, which is made out of polyester with four integrated polytetrafluoroethylene (PTFE) insulated AWG 32 strands (19 wires) and a diameter of 17 µm. The copper strands have a silver coating on the surface and no extra insulation around every wire. The tape consists of flexible (stretchable) areas and inflexible contact points. The flexible areas of the ribbon are achieved through the use of elastomers and the wrap-knitting technology. The ribbon was developed together with the company A MOHR Technische Textilien GmbH in Wuppertal and is now a commercial product of them. The isolation and small areas of the textile tape are removed locally at the contact points from both sides with a CO_2_ laser (Alltec LC300). A USS-4200 from MBR Electronics with different widths of tips (1, 3 and 5 mm) was used. The temperature is adjustable up to 500 °C; the ultrasonic power is variable between 4 W and 12 W at 60 kHz.

**Figure 2:**
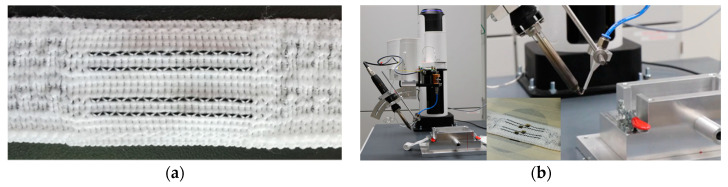
(**a**) Textile tape with flexible and stiff sections. The ribbon is 26 mm width and the position of the wires is axially symmetrical to the centreline. The outer wires have a distance of 12 mm and the inner wires a distance of 6 mm; (**b**) Pictures from the experimental setup, with the four-axis robot and the ultrasonic solder iron and the textile tape after CO_2_—Laser processing.

A four-axis industrial robot arm (Dobot M1) was programmed to automate and ensure reproducibility in the production of test samples (Figure 2a). In addition, a special holder for the textile tape and the PCB was manufactured. The PCBs have a size of 18 × 12 mm^2^. The ultrasonic soldering iron and the solder feed are carried out by the robot (Figure 2b). The soldering is done in two steps. First, the solder is melted at the tip of the soldering iron and, second, the tip of the soldering iron moves to the contact point. The tip of the soldering iron has a high carrying capacity for solder. We used three different solder alloys: Sn95.5Ag3.8Cu0.7, Sn99.7Cu0.3, and Sn97Ag3 without flux. These alloys are common in the electronics industry, in which flux-free solders are used.

### 2.3. Test Procedures

**Figure 3:**
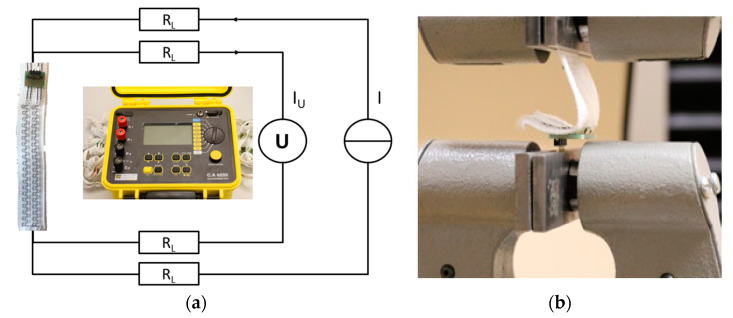
(**a**) Measuring setup for determining the contact resistances by using the four-wire measurement principle; (**b**) Peeling test set-up: The PCBs are fixed in the blank and pulled on the contacted textile bands with a speed of 100 mm/min [27].

To examine the samples, micrographs were first prepared and microscopically examined. The size and characteristics of the contact points can be examined under the microscope. By measuring the contact resistance, the quality of the joint can be examined. A four-wire measuring instrument is used to eliminate the line resistances (Figure 3a). The contact resistances are in the milliohm range [6]. High currents of up to 10 A are required to measure a measurable voltage drop at the contact point. Joints are usually characterized by a peel test. Peeling is usually the most unfavorable load direction for joints. In this case the peel strength is used to determine the mechanical strength of the joint for further processing. The force is applied vertically to the PCB (Figure 3b). Therefore, a load cell with a maximum force of 1000 N is used. The drawing speed is 100 mm/min. 10 samples were prepared for each test parameter.

## 3. Results and Discussion

### 3.1. Solder

**Figure 4:**
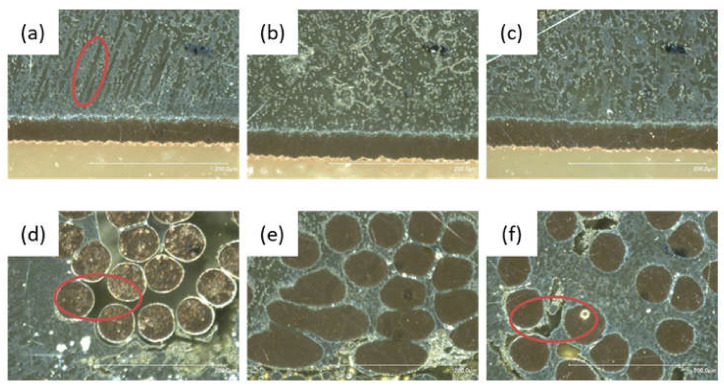
Microsections of various solder materials, (**a**,**d**) Sn95.5Ag3.8Cu0.7 with a needle-shaped microstructure in picture (**a**) marked with a red circuit, (**b**,**e**) Sn99.7Cu0.3, (**c**,**f**) Sn97Ag3; (**d**,**f**) not completely wetted electrical conductors also marked by red circuits.

Micrographs of the cross-section were made of all solder materials Sn95.5Ag3.8Cu0.7 (Figure 4a,d); Sn99.7Cu0.3 (Figure 4b,e); Sn97Ag3 (Figure 4c,f)). Sn99.7Cu0.3 (Figure 4e) shows significantly less pores compared to the samples (4d) and (4f). Sn95.5Ag3.8Cu0.7 (Figure 4a) has a needle-shaped micro-structure, which indicates that the metal alloy is brittle. The microstructures of Sn99.7Cu0.3 (Figure 4b) and Sn97Ag3 (Figure 4c) show a much more homogeneous picture.

**Figure 5:**
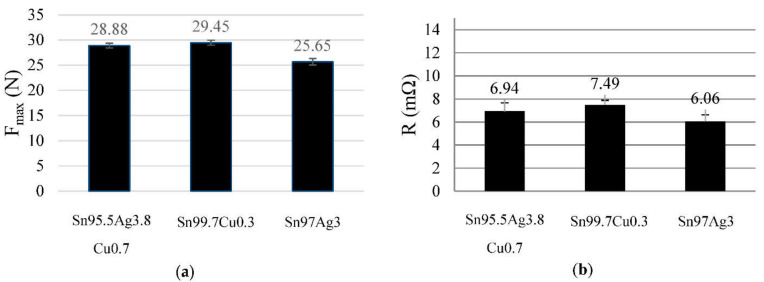
(**a**) Results of the peeling test and (**b**) resistances depending on the solder alloy.

This is also reflected in the results of the peeling test (Figure 5a). In the peeling test, the textile tape is pulled off at a 90° angle from the PCB. The contacting points usually fail one after the other. There are mainly two types of failure. One is the break outside the joining zone; the other is an adhesive breaking. In addition, other influencing variables and their effects were investigated. The used solder materials have effects on the contact resistance and the mechanical strength of the connection. Solder joints made of Sn97Ag3 had the lowest electrical resistance and low strength, Sn99.7Cu0.3 had the highest strength and also the highest electrical resistance (Figure 5b). In general, a higher silver content leads to a lower the resistance. In this case Sn95.5Ag3.8Cu0.7 represents a deviation from this. Furthermore, the degree of wetting of the electrical conductors has an impact on the resistance of the joints. Due to the high strength of Sn99.7Cu0.3 alloy and the hardly reduced contact resistance, the following tests were always carried out with this alloy. On the one hand the good wetting of Sn99.7Cu0.3 leads to high peel strength; on the other hand, the low silver ratio has not a high impact on the contact resistance.

### 3.2. Quantity of Solder

**Figure 6:**
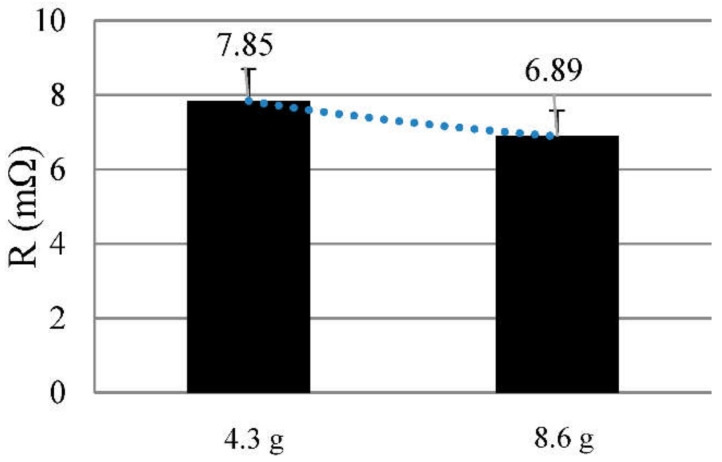
Contact resistance with different quantities of solder.

The amount of solder added has an influence on the electrical properties of the connection (Figure 6), while the mechanical properties in the peeling test remain the same. We used samples with 4.3 g and others with 8.6 g per contact point. Unfortunately, the automated solder feeding only allowed multiple quantities of this portion and 12.9 g was too much solder for one contact point. The more solder is used, the lower the contact resistance. Since the contact area has the same size for each sample, and all other parameter remains the same, the individual strands might be better wetted with more alloy. Regarding the high prices for flux-free solder alloys contact points with less solder might for most applications be sufficient.

### 3.3. Temperature

**Figure 7:**
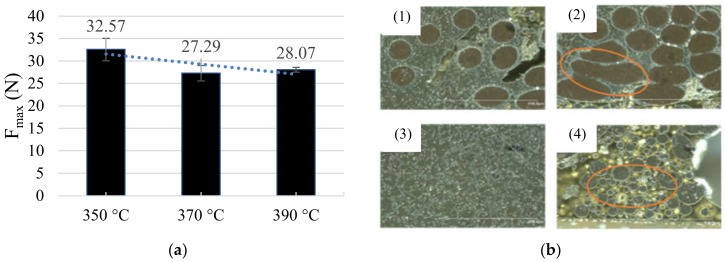
(**a**) Results of the peeling test with Sn99.7Cu0.3 depending on the solder tip temperature; (**b**) Microsections of Sn99.7Cu0.3 with different solder process temperatures: (1), (3) 350 °C and (2), (4) 390 °C; the red circuits show imperfections in the solder allow which reduce the mechanical strength of the connections.

The temperature at the soldering tip far above the liquidus temperature leads to lower strength. The samples produced with 370 °C show a bit lower strength than the samples made with 390 °C, but with a wider distribution (Figure 7a). A larger diameter of the soldering tip increases the heat capacity and thus facilitates faster melting of the solder. Furthermore, a larger soldering tip can lead to a resonance of the soldering tip with the solder. The different temperatures of the solder tip have no impact on the conductivity of the connection. The contact resistance is 7.65 mΩ +/− 0.23 mΩ. The strength increases by reducing the temperature. To further demonstrate the results, micrographs of the different samples were analyzed (Figure 7b). In comparison with contacting at 390 °C, contacting at 350 °C is optimized in two aspects. Due to the low heat input at 350 °C, the structure is more homogeneous and has fewer pores (Figure 7b (2)). Although there are some pores between the wire and the solder. This was also shown in the results of the tensile tests. The force during the peeling test of the 350 °C samples is greater than 32 N, which is about 20% more than with higher temperatures. The soldering process does not work at low working temperature like 320 °C, because the temperature does not liquefy the solder quickly enough.

### 3.4. Time of Soldering

**Figure 8:**
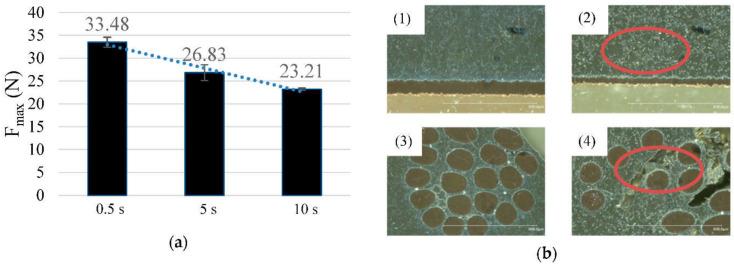
(**a**) The diagram shows the influence of the solder time and the mechanical strength. The lower the soldering time, the higher is the strength during the peeling test.; (**b**) Microsections of Sn99.7Cu0.3 samples, soldered with different ultrasonic solder times: (1), (3) 0.5 s solder time; (2), (4) 10 s solder time. The red circuits show imperfections in the allow after a longer ultrasonic soldering time.

Additionally, the influence of time on ultrasonic soldering was investigated. It was found that a longer soldering time in combination with the ultrasonic application has a negative influence on the strength of the samples during the peel test (Figure 8a,b). Samples that were treated for only 0.5 s showed a more than 40% higher peel strength than samples that were soldered for 10 s. So, the removal of foreign layers by ultrasonic application might be a very fast process. 

Although the application of ultrasound causes larger grains to break up, thus reducing the crystal grains of the alloy, the higher heat input inevitably leads to a coarsening of the crystal grains [28]. In addition, the longer temperature effect during ultrasonic soldering leads to overheating of the solder joints, as well as to oxidation and charring. This reduces the mechanical properties of the solder joints [29]. However, the contact resistance between the samples does not differ.

### 3.5. Ultrasonic Power

**Figure 9:**
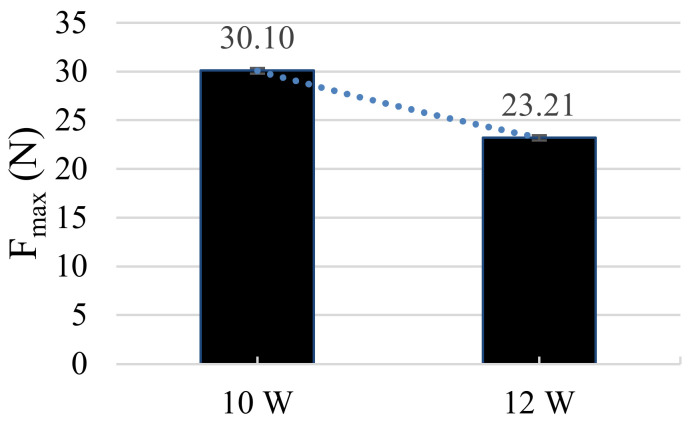
The Diagram illustrates a correlation between power and mechanical strength. A reduced ultrasonic power of 10 W leads to a higher mechanical strength.

The power mainly influences the variation of the amplitude during the movement of the solder tip under ultrasonic waves, and the adjustment range of our soldering machine is from 4 to 12 W. During our investigations, we found that the power has an important influence on the soldering process, especially for the melting process of the solder. At high power, the solder was quickly homogenized after melting and formed into droplets. This allows the tip to carry and feed more solder. During the soldering process, the spread on the soldering plate at high power was significantly better than at low power, which helps to increase the solder joint strength. However, there was also a maximum amount of power at which the strength was significantly reduced again. At an output of 10 W, the joint strength was 25% higher than at 12 W (Figure 9), because an increased ultrasonic power ensures a more homogeneous micrograph at the contact point. The contact resistances are almost identical. Due to the choice of the test device, no higher power than 12 W could be tested. Soldering was not possible at too low power levels because the solder did not spread fast enough and the impurity layers may not be removed.

### 3.6. Pretreatment

**Figure 10:**
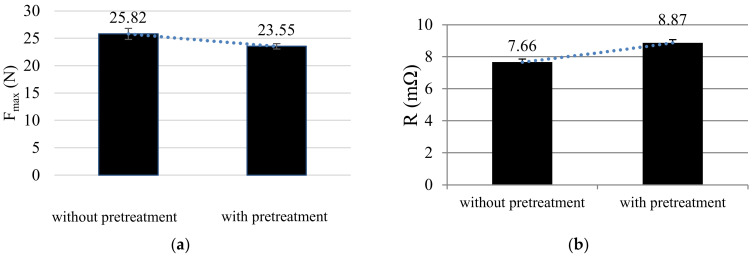
(**a**) With the reduced tensile strength during the peeling test after pretreatment; (**b**) with the increased contact resistance after pretreatment with acetone.

We also investigated the effects of pretreatment or surface with cleaners. We found that the additional pretreatment of the contact points with acetone has a negative influence on both the tensile strength and the electrical resistance (Figure 10). The tensile strength during the peeling test decreased from 25.5 N to 23.5 N, which means around 10%. In X-ray Spectroscopy (XPS) tests for surface cleaning, it was proven that acetone or other cleaning agents leave (carbon) residues on surfaces that negatively influence subsequent coating processes [30].

Related to our test setup and our equipment possibilities, we found the following soldering parameters as a compromise between shear strength and contact resistance: Sn99.7Cu0.3 at 350 °C, an ultrasonic working time of 0.5 s and a power of 10 W (Table 1). The results are based on the fact that the process is quite fragile and needs automation to be reliable. Using this technique manually leads to a many poor connections. A lot more parameter have been tried, but they were not successful.

## 4. Conclusions

This article focuses on the development of a contacting method by means of a partially automated ultrasonic soldering system to integrate PCB components on smart textiles. Three solder alloys were chosen: Sn95.5Ag3.8Cu0.7, Sn97Ag3 and Sn99.7Cu0.3 without flux. Subsequently, the mechanical and electrical properties of the soldered connection were analyzed as a function of the soldering parameters. Therefore, a contact resistance measurement and a peeling test of the connections were conducted.

We analyzed the influence of the solder alloy itself, the quantity of solder, the solder temperature, the time of soldering, the ultrasonic power and the pretreatment of the surface before soldering as a function of the contact resistance of the connections and their strength during the peeling test. One of the main problems is the durability of the electronics. The investigations of the peeling test showed that connections with solder made of Sn99.7Cu0.3 yield higher strengths due to connections with Sn95.5Ag3.8Cu0.7 or Sn97Ag3. The contact resistances ranged from 6 to 8 mΩ. For this reason, the alloy with the highest peel strength was chosen: Sn99.7Cu0.3. The quantity of solder also has only a limited influence on the strength and contact resistance, thus we recommend not to choose more solder alloy than necessary. Further, the solder temperature is also a very important influencing factor. In addition, too high or too low working temperature damages the contact. The ultrasonic power and the ultrasonic process time have a great influence on the formation of the microstructure. The shorter the ultrasonic treatment time during the soldering process, the better the properties of the solder connection. The reason for this is the better homogeneous microstructure with only few defects. Overall, the ultrasonic welding process looks very promising for the integration of electronics to textiles, because with the dispense of flux in the solder alloy less solder draws into the litz wire, which reduces the embrittlement of the wire. We found the following optimal soldering parameters as a compromise between shear strength and contact resistance: Sn99.7Cu0.3 at 350 °C and an ultrasonic working time of 0.5 s and a power of 10 W.

## Figures and Tables

**Table 1:** Results from this investigation.

Solder Material	Quantity	Time	Temperature	Power
Sn99.7Cu0.3	4.3 g	0.5 s	350 °C	10 W

## Data Availability

Data sharing not applicable.
